# Perioperative Dynamics of TLR2, TLR4, and TREM-1 Expression in Monocyte Subpopulations in the Setting of On-Pump Coronary Artery Bypass Surgery

**DOI:** 10.1155/2013/817901

**Published:** 2013-03-17

**Authors:** A. S. Golovkin, V. G. Matveeva, I. V. Kudryavtsev, M. N. Chernova, Y. V. Bayrakova, D. L. Shukevich, E. V. Grigoriev

**Affiliations:** ^1^Research Institute for Complex Issues of Cardiovascular Diseases under the Siberian Branch of the Russian Academy of Medical Sciences, 6 Sosnovy Boulevard, Kemerovo 650002, Russia; ^2^Research Institute for Experimental Medicine under the NorthWest Branch of the Russian Academy of Medical Sciences, Saint Petersburg, Russia

## Abstract

Hypercytokinemia plays a key role in the pathogenesis of systemic inflammatory response syndrome (SIRS). Monocytes are the main source of cytokines in the early inflammatory phase. Simultaneous stimulation of toll-like receptors (TLRs) and triggering receptor expressed on myeloid cells (TREM-1) activating receptor on monocytes results in the amplification of the inflammatory signal and multiple increase in proinflammatory cytokine production. The dynamics of those receptors expression on monocyte surface of patients with uncomplicated SIRS course followed coronary artery bypass surgery (CABG) was studied. The increase in TLR2 and TREM-1 expression on the first day after CABG induces proinflammatory and amplification potentials of monocytes in that period. The decrease in TLR2 surface expression on the seventh day compared to the preoperative values can be regarded as a mechanism limiting inflammatory response. The highest level of TLR2, TLR4, and TREM-1 surface expression was observed in CD14^hi^CD16^+^ monocyte subpopulation, confirming its proinflammatory profile.

## 1. Introduction

 The early postoperative period in CABG patients is associated with systemic inflammatory response syndrome (SIRS), which is complicated with multiple organ dysfunction and high mortality in 5%–16% of cases (EACTA 2007 data) [1, 2]. Significant progress has been made over the last decade in understanding the pathophysiology of critical conditions. SIRS is still being investigated, and its criteria and clinical features of its course for different conditions and diseases are being specified and elaborated [3]. The success of on-pump heart surgery often depends on the prompt intensive treatment and prevention of SIRS complications in the postoperative period. Therefore, SIRS course and criteria as well as the ways of predicting its complications are of a great interest in this group of patients. 

Today, the leading role of the immune system in the SIRS development has been confirmed. Hyperproduction of proinflammatory cytokines, oxidative stress, and protease storm are essential pathogenetic components of the hyperergic phase of systemic inflammation. The congenital immune system is inseparably linked to those processes [3].

Maintenance of certain antigenic composition in the body and detection and removal of exogenous (microorganisms) and endogenous (malignant cell transformations) hostile macromolecules used to be the main functions of the congenital immune system. However, nowadays, its functions are borne in on to be wider, because it is involved in regulating internal environment, suffering from any disorders, to maintain stable condition, especially after the impact produced by the damaging factors, not related with microorganisms. Those factors include mechanical and reperfusion injuries, ischemia, various burns, UV rays, and radiation [4–6]. Adverse environmental effects of enough strength and duration cause unprogrammed cell death and immediate release of endogenous molecules or alarmines, which are normally inside the cells, into the intracellular environment. Alarmines activate the congenital immune system inducing inflammation and promoting tissue recovery [5, 6].

Currently, cellular endogenous molecules (heat-shock proteins, mitochnodrial formyl peptides, uric acid crystals, defensins, HMGB-1 cytokines, etc.) as well as fragments of damaged extracellular matrix (hyaluronan, fibronectin, heparin sulfate, biglycan, tenascin-C, fibrinogen, etc.) are classified as alarmines [4–6].

It is worthy to note that the congenital immune system employs an almost similar complex of defensive responses in response to any damage [6]. This is mostly due to surface toll-like receptors (TLRs) that can recognize both extracellular highly conserved microorganism structures or PAMPs (pathogen-associated molecular patterns) and alarmins further activating transcription factors of the same proinflammatory genes coding the production of proinflammatory regulatory substances, including cytokines and chemokines [4, 5].

TLR4 ligands can be lipopolysaccharides (LPS) of gram-negative bacteria as well as heat shock proteins (HSPs) [7], HMGB-1 [8], hyaluronan and its fragments, biglycan [4, 5], uric acid crystals [9], heparin sulfate [5], calcium-binding protein A (S100A) [6], and so forth. TLR2 recognizes cell-wall components such as peptidoglycans, lipoteichoic acid (LTA) of gram-positive bacteria, some components from mycobacteria, and zymosan from yeast cell wall as well as HSPs, HMGB1, hyaluronan and its fragments, and biglycans [5]. 

Developing an adequate protective effect in case of a massive injury requires the amplification of PRR signals involving other molecules and receptors [10]. Such amplifying potential is a property of triggering receptor expressed on myeloid cells (TREM-1) activating receptor [11, 12].

Isolated TREM-1 binding to anti-TREM-1 antibodies causes intensive and immediate activation of all the effector mechanisms [11]. TREM-1 activation in neutrophils is characterized by a rapid degranulation, respiratory stimulation, G-CSF and IL-8 secretion, and to a lesser extent with the phagocytic activity [13]. Monocytes respond to such an impact by the increases in proinflammatory cytokines (TNF*α*, IL-1*β*, IL-6, and M-CSF) and chemokines (IL-8, MCP-1, MCP-3, and MIP-1*α*) production and the decrease in anti-inflammatory IL-10 secretion [11, 14]. 

However, simultaneous stimulation of TLRs and TREM-1 induces the amplification of inflammatory signals with the further synergetic cytokine production. This effect is well studied in TLR2 and TLR4. The impact of anti-TREM-1 antibodies on monocytes in the presence of LPS or LTA results in 5- or 20-fold increase in TNF*α*, IL-6, GM-CSF, and MCP-1 production, compared with the separate impact of each stimulator, and almost total (80%–90%) inhibition of IL-10 production [11, 12].

The active search for a natural TREM-1, which has not yet been exactly identified, is underway. However, its presence was registered in the blood serum of some septic patients [15]. HMGB1 and HSP70 alarmins, found in the lysate of necrotic cells, were suggested to be ligands for TREM-1 according to the obtained indirect evidence [16]. 

The presence of TREM-1 ligand in the blood, while TLRs are activated, promotes a multiple increase in proinflammatory cytokine production. Cytokines have a regional effect; however, entering the blood flow in case of insufficient anti-inflammatory resistance mechanisms, can cause a systemic inflammatory response syndrome [3, 17].

The population of monocytes, considered to be the main cytokine-producing cells in the congenital immune system, is not homogenous. According to the level of surface CD14 and CD16 expression, 3 monocyte subpopulations are classified: CD14^hi^CD16^−^, CD14^hi^ CD16^+^, and CD14^dim^CD16^+^ [18]. All those subpopulations differ by their functional activity, the spectrum of produced cytokines, and the number and the expression intensity of surface receptors, regulating various functions in the human body [19]. Experimental and clinical studies have proved a dominating role of CD14^hi^CD16^+^subpopulation in septic processes and SIRS [20]. 

Because of enough compensatory abilities of inflammation resistance factors, the hyperergic phase in patients with uncomplicated SIRS course, developing during the surgery and in the immediate postoperative period hours, provokes the recovery phase. Such SIRS course is supposed to be the most favorable. 

In order to predict possible SIRS complications following the direct on-pump myocardial revascularization, it is important to evaluate immunological characteristics of a favorable SIRS course in this group of patients. We suggest the level of TREM-1, TLR2, and TLR4 surface expression to determine their potential to the inflammatory response amplification. 

This study aimed at evaluating the consistent patterns of TREM-1, TLR2, and TLR4 surface expression dynamics on monocyte subpopulations of patients with uncomplicated postoperative period who underwent on-pump coronary artery bypass surgery. 

## 2. Materials and Methods

A number of 25 coronary artery disease patients with angina pectoris CCSC II-III and chronic heart failure (CHF) I-IIA (NYHA II-III) aged 47–70 years old were enrolled in this study. The exclusion criteria were as follows: combined coronary artery and valvular heart disease, acute infection and chronic infection exacerbation, cancer, and postoperative surgical complications. All the patients had direct myocardial revascularization performed with standard cardioplegia and non-pulsatile cardiopulmonary bypass (CPB). Intravenous general anesthesia with fentanyl and midazolam was induced. Homotypic hypertonic hyperoncotic perfusate was used for CPB with the initial priming volume for the heart-lung machine. Cold blood cardioplegia was used, and cardioplegia was delivered anterogradely. The bypass time was 88 min (75–105 min) and the aortic cross-clamp time was 57 min (48–61 min). 

SIRS was assessed in all the patients according to 4 criteria, approved at the Joint Conference in Chicago, and had score 2-3 at day 1. 

To study monocytes, the blood was taken from the peripheral vein into the vials with K_3_EDTA before surgery and at day 1 and 7 after the surgery. The staining was done in accordance with the protocol of the manufacturing companies using monoclonal CD16-FITC, CD14-APC or CD14-PE antibodies (Beckman Coulter, USA), TLR2-APC, TLR4-PE (eBioscience, USA), and TREM-1-PE (R&D, USA). The control consisted in administering the same amount of antibodies of the relevant isotope control. The cells were incubated with the antibodies at 4°C during 30 min in the dark. Erythrocyte lysis was done with the BD FACS lysing solution (BD Bioscience, USA). After 10 min incubation, the cells were once washed with the excess of the PBS. The obtained sediment was suspended in the PBS. 

**Figure 1:**
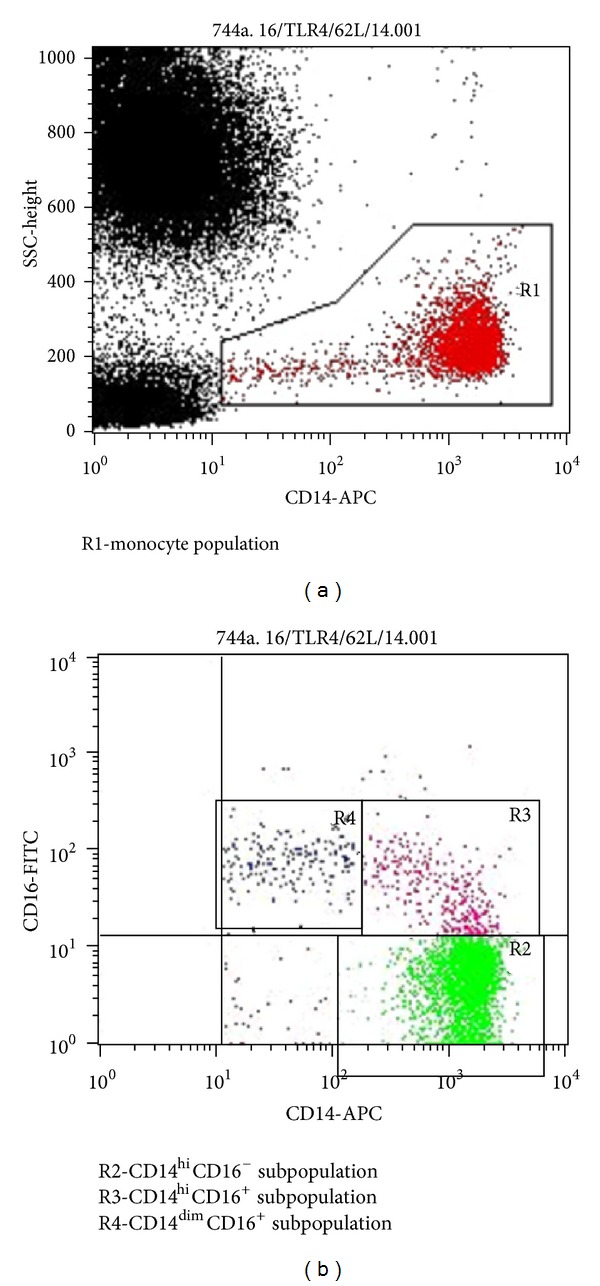
Monocytes subpopulations.

**Figure 2:**
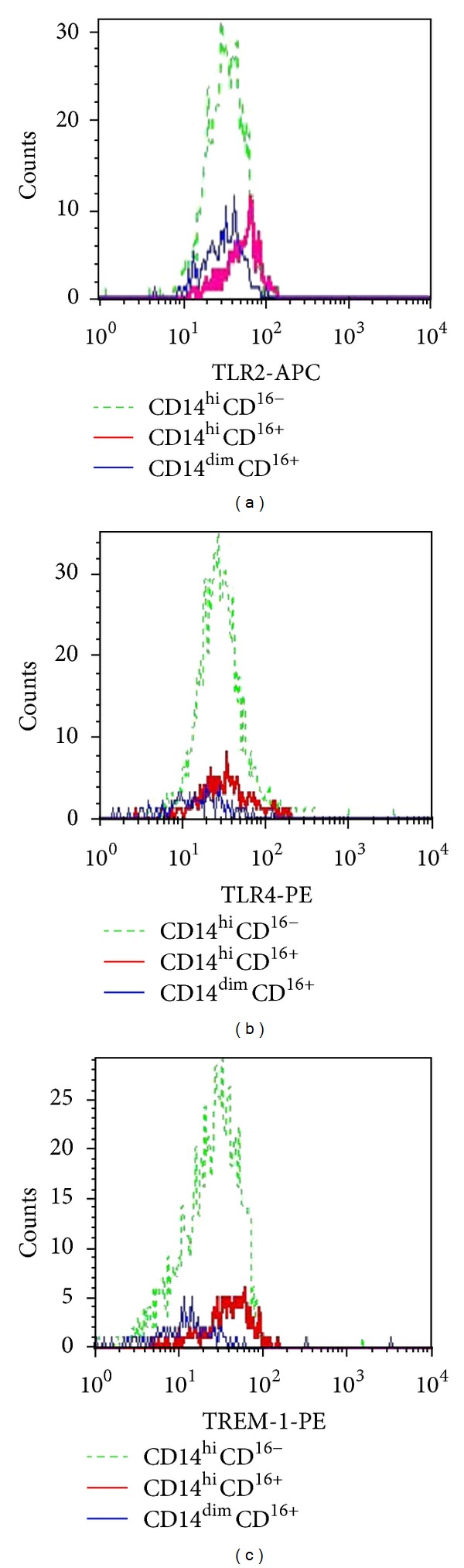
The mean intensity of fluorescence of TLR2, TLR4, and TREM-1 surface expression.

Cytofluorometry was done using FACSCalibur flow laser cytometer (Becton Dickinson, USA). CellQuestPro with the same settings was used for all the tests. Not less than 3000 monocytes were analyzed in every sample. CD14 was used to extract a monocyte population together with side scattering (SSC). According to the level of CD14 and CD16 expression, the monocytes were divided into three subpopulations: CD14^hi^CD16^−^, CD14^hi^CD16^+^, and CD14^dim^CD16^+^ (Figure 1). The level of TLR2, TLR4, and TREM-1 surface expression was evaluated separately for each subpopulation according to the mean intensity of fluorescence (MIF) using geometric mean values (Figure 2).

The statistical analysis was conducted using STATISTICA 6.0 software package. The Wilcoxon test was used to evaluate the significance of differences. The data were presented as a median and interquartile range (IQR). 

## 3. Results

### 3.1. Evaluating Preoperative Levels of TREM-1, TLR2, and TLR4 Surface Expression in Monocyte Subpopulations (MIF Based)

Significant differences in the levels of TLR2, TLR4, and TREM-1 surface expression in the monocyte subpopulations were found (Table 1). The maximum MIF values for those receptors were registered in the monocytes with CD14^hi^CD16^+^phenotype (Figure 2). CD14^hi^CD16^+^subpopulation had the lowest MIF values for TLR4 and TREM-1 receptors as well as similar TLR2 surface expression (MIF based). 

Then, the dynamics of surface expression was studied separately for each receptor. 

### 3.2. Evaluating Perioperative Levels of TLR2 Surface Expression in Monocyte Subpopulations in Patients Undergoing Direct On-Pump Myocardial Revascularization

There was an increase in the density of TLR2 surface expression (MIF based) in the monocytes with CD14^hi^CD16^+^ and CD14^dim^CD16^+^ phenotypes compared to the preoperative values at day 1 after the surgery (Table 2). At day 7 after the surgery, there was less TLR2 MIF in all the monocyte subpopulations compared with day 1, with MIF values being lower than those before the surgery. 

### 3.3. Evaluating Perioperative Levels of TLR4 Surface Expression in Monocyte Subpopulations in Patients Undergoing Direct On-Pump Myocardial Revascularization

At day 1 after the surgery, there was a decrease in TLR4 MIF in the monocytes with CD14^hi^CD16^−^ phenotype compared with the preoperative values. At day 7 after the surgery MIF was also lower than initial preoperative values (Table 3). There was no TLR4 MIF dynamics in other subpopulations at the analyzed time points. 

### 3.4. Evaluating Perioperative Levels of TREM-1 Surface Expression in Monocyte Subpopulations in Patients Undergoing Direct On-Pump Myocardial Revascularization

The dynamics of TREM-1 surface expression in all the monocyte subpopulations was unidirectional (Table 4). At day 1 after the surgery, TREM-1 MIF increased in comparison with preoperative values. At day 7 after the surgery, TREM-1 expression was lower and did not differ from the preoperative level.

### 3.5. Evaluating Perioperative Levels of CD14 Surface Expression in Monocyte Subpopulations in Patients Undergoing Direct On-Pump Myocardial Revascularization

CD14 MIF in the monocytes with CD14^hi^CD16^−^ phenotype decreased at day 1 after the surgery compared to the preoperative values, and by day 7 that decrease had been postponed in all the subpopulations (Table 5). At day 7 of the postoperative period, there was less CD1 MIF in CD14^hi^CD16^+^ and CD14^dim^CD16^+^ subpopulations and it increased in CD14^hi^CD16^−^ subpopulation. 

### 3.6. Evaluating the Correlations between CD14, TLR2, and TLR 4 Mean Intensity of Fluorescence in Different Monocyte Subpopulations

A positive correlation between the MIF of CD14 and TLR2 receptors in CD14^hi^CD16^−^ subpopulation at all analyzed time points was found (Table 6). CD14^hi^CD16^+^ subpopulation reported strong correlation between the surface expression levels of those receptors at day 1 after the surgery; CD14^dim^CD16^+^ monocytes found a moderate correlation before the surgery. 

The MIF of CD14 and TLR4 receptor correlation was registered only at day 1 after the surgery in CD14^hi^CD16^+^ monocytes. 

## 4. Discussion

Monocyte subpopulations have different functional characteristics proved experimentally and clinically. 

The most numerous CD14^hi^CD16^−^ subpopulation, which normally makes up 90%–95% of all the blood monocytes, is characterized by active chemokine production (IL-8, CCL2, and CCL3) and marked phagocytic and microbicidal activity but used to have low proinflammatory cytokines production [21].

A minor CD14^hi^CD16^+^ subpopulation unlike CD14^hi^CD16^−^ monocytes has a limited capacity to respiratory activation and phagocytosis; however, it actively produces proinflammatory cytokines (TNF*α*, IL-1*β*, and IL-6) [21, 22]. According to the above mentioned characteristics, CD14^hi^CD16^+^monocytes are called “proinflammatory.” The previous clinical studies reported its increase in patients with systemic inflammatory response syndrome (sepsis, endotoxicosis) [20].

CD14^dim^CD16^+^ monocytes have a high affinity to endothelium and high migration activity; therefore, there is only up to 25% of this subpopulation in the circulating blood [23]. In response to stimulation and those cells do not produce reactive oxygen forms and demonstrate low phagocytic capacity, low myeloperoxidase, lysozyme, and proinflammatory cytokine production; however, they constitutively produce IL-1RA. Therefore, in terms of phagocytic and microbidic activity and cytokine production, CD14^dim^CD16^+^ monocyte profile is sometimes called “anti-inflammatory” [21]. In case of ischemic myocardial injury, this subpopulation is supposed to take part in tissue reparation, involving fibroblasts, stimulating angiogenesis and collagen buildup [24].

Preoperative levels of TLR2, TLR4 and TREM-1 surface expression before the surgery were studied. CD14^hi^CD16^+^ monocyte surface showed the highest expression of TLR2, TLR4, and TREM-1 confirming proinflammatory properties of these monocytes (Figure 2) because the stimulation of these receptors results in the active production of proinflammatory cytokines. Low surface expression of TLR4 and TREM-1 was registered in CD14^dim^CD16^+^ subpopulation confirming its anti-inflammatory profile. 

Uncomplicated SIRS in the immediate postoperative period is not associated with infection. It is considered to develop due to a massive alarmin release in response to ischemia reperfusion, mechanical injury, and operative stress. Identification of alarmins by the congenital immune system receptors, similar to bacterial pathogens, causes a cascade activation of proinflammatory genes and cell stress [3]. 

Nowadays, TLR2 receptors are inseparably linked with the progression of ischemic and reperfusion myocardial injuries [25]. TLR2 express cells of various compartments, involved in ischemia-reperfusion injury: cardiomyocytes, endotheliocytes, and leukocytes. Experimental data reported TLRs activity in various compartments to be associated with myocardial ischemia-reperfusion injury manifestations. Impaired cardiac contractility is associated with the increase in TNF*α*, IL-1*β* in the myocardium and cardiomyocyte TLR2 activation [26]. Endotheliocyte and leukocyte TLR2s are involved in endothelial dysfunction development, manifested by “no-reflow” phenomenon [26]. Cardiomyocyte death and infarct zone development in fatal ischemia-reperfusion is caused by leukocyte TLR2 activation. Experiments with mice leukocyte TLR2 inhibition demonstrated the reduction in the infarct size and improvement in cardiac function due to the decrease in inflammatory response and cardiomyocyte apoptosis [27]. 

In vitro experiments with LPS and LTA reported increases in mRNA and monocyte surface TLR2 and TLR4 expression during the first stimulation hours and the decrease in expression if the stimulation continued up to 20 hours and more [28–30]. One of the mechanisms of such decrease in monocyte surface TLR2 and TLR4 expression can be related to the internalization of LTA/CD14/TLR2 or LPS/CD14/MD2/TLR4 complex and its rapid transition into the Golgi apparatus, resulting in signaling limitation and further antigen disposal and/or presentation [30–32]. TLR2 and TLR4 stimulation by bacterial pathogens and alarmins, activating similar intracellular signaling mechanisms with further cytokine synthesis, has similar mechanisms of expression and internalization of those receptors. 

The dynamics of TLR2 and TLR4 surface expression in monocyte subpopulations of patients with uncomplicated postoperative period after the direct myocardial revascularization was studied. The increase in TLR2 expression was observed in all the monocyte subpopulations immediately after the surgery, suggesting the stimulation of its receptors and its significance in the early postoperative period. Activation of adaptive mechanisms, limiting inflammatory response in the late postoperative period, was registered by the decrease of TLR2 expression compared with preoperative values. 

The positive correlation of CD14 and TLR2 fluorescence intensity in CD14^hi^CD16^−^ and CD14^hi^CD16^+^ monocytes can be regarded as an indirect evidence for ligand/TLR2/CD14 complex cooperation and internalization, because, while the complex is being internalized, the changes in CD14 and TLR2 surface expression level should be proportional or almost proportional. 

TREM-1 surface expression can determine the amplifying potential of the cells, and we have evaluated its postoperative dynamics in patients with uncomplicated SIRS who have undergone CABG. An increase in TREM-1 antibody fluorescence intensity on the surface of all the monocyte subpopulations at day 1 after the surgery indicated the increase in the monocyte amplifying inflammatory potential in that period. Interestingly, a more significant 1.5-fold increase was observed in “proinflammatory” CD14^hi^CD16^+^ subpopulation. 

Thus, patients with uncomplicated postoperative period after on-pump CABG reported increases of proinflammatory and amplifying potentials of monocytes at day 1 after the surgery due to higher TLR2 and TREM-1 surface expression. Inflammation limiting mechanisms are activated and manifested by lower TLR2 surface expression in the late postoperative period compared to the preoperative values. 

The highest levels of TLR2, TLR4, and TREM-1 surface expression were observed in CD14^hi^CD16^+^ subpopulation, which confirms its proinflammatory profile. 

## Figures and Tables

**Table 1:** Preoperative surface expression levels of the studied receptors in monocyte subpopulations.

	TLR2 (MIF)			TLR4 (MIF)			TREM-1 (MIF)		
	Median	IQR	Wilcoxon	Median	IQR	Wilcoxon	Median	IQR	Wilcoxon
			*P *			*P *			*P *
CD14^hi^CD16^−^	45.6	15.4		32	13.6		22.6	10.9	

CD14^hi^CD16^+^	75.2	26.5	<0.0001*	46.6	20	<0.0001*	30.3	11.3	<0.0001*

CD14^dim^CD16^+^	49.3	15.8	= 0.583*	16.6	7.7	<0.0001*	16.7	5.1	<0.0001*
			<0.0001**			<0.0001**			<0.0001**

*Compared with CD14^hi^CD16^−^.

**Compared with CD14^hi^CD16^+^.

**Table 2:** Dynamics of TLR2 surface expression in monocyte subpopulations.

	Before the surgery		1 day after the surgery			7 days after the surgery		
	Median	IQR	Median	IQR	Wilcoxon	Median	IQR	Wilcoxon
					*P *			*P *
CD14^hi^CD16^−^	45.6	15.4	48.1	13.4	= 0.304*	30.3	11.2	<0.0001*
								<0.0001**

CD14^hi^CD16^+^	75.2	26.5	81.2	23.6	<0.05*	47.3	22.3	<0.0001*
								<0.0001**

CD14^dim^CD16^+^	49.3	15.8	52.3	10.1	<0.0001*	35.5	13.7	<0.0001*
								<0.0001**

*Compared with preoperative values.

**Compared with day 1 values.

**Table 3:** Dynamics of TLR4 surface expression in monocyte subpopulations.

	Before the surgery		1 day after the surgery			7 days after the surgery		
	Median	IQR	Median	IQR	Wilcoxon	Median	IQR	Wilcoxon
					*P *			*P *
CD14^hi^CD16^−^	32.0	13.6	27.3	7.5	<0.02*	29.0	15.5	<0.05*
								= 0.511**

CD14^hi^CD16^+^	46.6	20	41.9	17	= 0.753*	40.0	15.5	= 0.078*
								= 0.051**

CD14^dim^CD16^+^	16.6	7.7	14.9	8.3	= 0.648*	15.2	6.2	= 0.465*
								= 0.627**

*Compared with preoperative values.

**Compared with day 1 values.

**Table 4:** Dynamics of TREM-1 surface expression in monocyte subpopulations.

	Before the surgery		1 day after the surgery			7 days after the surgery		
	Median	IQR	Median	IQR	Wilcoxon	Median	IQR	Wilcoxon
					*P *			*P *
CD14^hi^CD16^−^	22.6	10.9	25.4	13.5	<0.0001*	22.1	11.3	= 0.326*
								<0.0001**

CD14^hi^CD16^+^	30.3	16.2	42.3	20.9	<0.0001*	29.5	15.4	= 0.589*
								<0.0001**

CD14^dim^CD16^+^	16.7	5.1	26.4	13.3	<0.0001*	15.9	7.5	= 0.276*
								<0.0001**

*Compared with preoperative values.

**Compared with day 1 values.

**Table 5:** Preoperative dynamics of CD14 surface expression in monocyte subpopulations.

	Before the surgery		1 day after the surgery			7 days after the surgery		
	Median	IQR	Median	IQR	Wilcoxon	Median	IQR	Wilcoxon
					*P *			*P *
CD14^hi^CD16^−^	1429.5	280.5	755.8	300.9	<0.0002*	957.6	287.7	<0.0002*
								<0.05**

CD14^hi^CD16^+^	1315.2	358.7	1116.9	468.3	= 0.295*	965.9	174.2	<0.0003*
								<0.006**

CD14^dim^CD16^+^	82.3	31.9	98.1	42.1	= 0.126*	72.9	23.3	<0.05*
								<0.05**

*Compared with preoperative values.

**Compared with day 1 values.

**Table 6:** Correlations between CD14 and TLRs MIF.

Subpopulations	CD14 MIF					
	Before the surgery (MIF)		1 day after the surgery (MIF)		7 days after the surgery (MIF)	
	TLR2	TLR4	TLR2	TLR4	TLR2	TLR4
CD14^hi^CD16^−^	**0.56**	0.07	**0.83**	0.37	**0.60**	−0.29

CD14^hi^CD16^+^	0.27	0.15	**0.80**	**0.47**	0.27	−0.06

CD14^dim^CD16^+^	**0.46**	−0.05	0.4	0.02	−0.03	0.22

Significant correlations are in bold, *P* < 0.05.
